# A Computerized Tool to Support Survivorship Care

**Published:** 2013-01-01

**Authors:** Michele R O’Brien, Kimberly K. Ness, Chara J. Anderson, Mark D. Sborov, Jackie D. Foster

**Affiliations:** From Minnesota Oncology, Edina, Minnesota

Cancer survival has improved as a result of recent advances in treatment and detection. There are currently 12 million cancer survivors in the United States; by 2020 that number is expected to reach 20 million (National Cancer Institute, 2011). The growing numbers of survivors include not only patients who are cured, but also patients who are living with advanced cancer.

Cancer survivorship is an immense topic that includes care coordination, surveillance, long-term and late effects, and psychosocial effects (Grant, Economou, & Ferrell, 2010). National standards, guidelines, and recommendations add to the already complex scope of survivorship care. These include the American College of Surgeons Commission on Cancer (CoC) 2012 standards for patient-centered treatment, Medicare’s "meaningful use," and the Institute of Medicine’s recommendation for shared decision-making (Institute of Medicine, 2001). Specifically, the CoC standards include patient navigation, distress measurement, and treatment summaries (Commission on Cancer, 2011).

Collectively, the scope of survivorship care and national requirements creates challenges for patients, caregivers, and medical practices. What is the most practical approach to tackle these challenges? As standards are mandated, how do we create tools that cross utilization lines? One answer is to use a computerized hand-held tablet to support many components of survivorship care (see Figure 1 for an overview of the tablet’s role in gathering data in practice).

**Figure 1:**
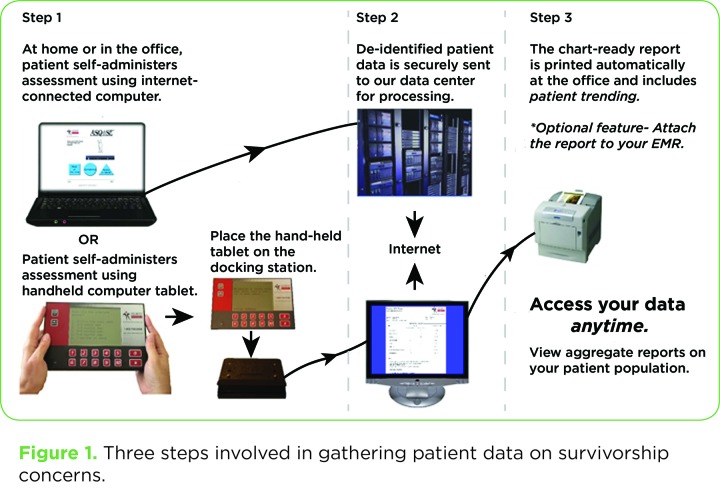
Figure 1. Three steps involved in gathering patient data on survivorship concerns.

Minnesota Oncology’s survivorship team reviewed survivorship components, national standards, and programmatic needs (Sborov & O’Brien, 2012). To support advanced practitioners (APs) providing survivorship care, we designed a tablet-based questionnaire and a combined report that serves both clinical and programmatic functions (Hunter, 2012). In addition, incorporating the tablet supports the practice in meeting and measuring national standards for distress screening, navigation, meaningful use, and shared decision-making.

## Incorporating a Tablet Into Survivorship Care

Each patient is asked to complete a questionnaire on a tablet prior to every survivorship visit, requiring approximately 7 to 10 minutes. The components of the questionnaire (preloaded by the survivorship team) include the Functional Assessment of Chronic Illness Therapy (FACIT), as well as items related to patient concerns and patient satisfaction. Responses are downloaded to a computer database. A report that is uniquely designed for the patient and the AP is generated. The patient and AP each receive a printed copy of the report at the onset of the visit. This real-time report becomes the framework for the survivorship visit. At the end of the visit, the AP report is scanned into the electronic medical record.

The FACIT, a validated quality-of-life (QOL) tool, evaluates four domains of a patient’s well- being: physical, functional, social, and emotional (Webster, Cella, & Yost, 2003). The report includes standardized QOL scores for each domain on a 0 to 100 scale. Higher scores signify higher QOL. The QOL scores are a screening tool for the AP. There is no evidence that a specific score requires a specific intervention, but the scores help the AP define what is important to the patient. Then the AP can apply interventions based on skilled assessment and counseling to help improve the patient’s QOL as he or she defines it.

There is a section for the patient to identify current concerns, which helps the AP characterize the sources of distress. Thus, the tablet supports the standard for distress screening. The patient receives a report that links each specific concern to relevant local and national resources. The AP report provides a list of referrals that are appropriate for the patient’s concerns. During the visit, the AP uses the report to guide the patient, thus meeting the standard for navigation. The AP delegates follow-up on interventions, referrals, and patient status to RNs as needed. Subsequent survivorship visits are an opportunity to follow up on the patient's concerns.

## Advantages and Usefulness

An advantage of using a computerized tablet is that it records the trends in a patient’s QOL. It is common for QOL concerns and distress to change throughout a patient’s cancer journey. Monitoring the trend assists the AP or physician when discussing interventions and treatment decisions that will impact the patient’s QOL.

Furthermore, the tablet’s usefulness extends beyond the immediate patient encounter. One of the questions in the satisfaction survey section of the questionnaire covers patient involvement in health-care decisions. This helps our practice address shared decision-making and set goals for improvement. In addition, measuring and reporting patient satisfaction increases acceptance of the tablet and survivorship visit within the organization.

Aggregate data about QOL and concerns for the entire clinic are stored, and reports are accessible. Cumulative data support ongoing evaluation of care quality and provide insight for system-wide improvements. Commonly identified patient concerns indicate what services or referral lines are needed to meet patient needs.

It is important to consider feasibility and logistics in a busy clinic setting before adopting a new technology. Significant time is required initially to design reports that meet clinical needs. Knowledge of the community is necessary to link patient concerns with resources and referrals. Medical assistants can be trained to administer the tablet-based questionnaire. Technologic support and a designated computer for downloading and reviewing reports are necessary.

## Tool Limitations and Future Directions

One limitation of the tablet is that it does not assist with treatment summaries or care plans, including screening or health promotion recommendations. However, we see this as more of a limitation of our electronic medical record. The tablet is designed to support the APN with distress management and patient navigation, two large components of survivorship care that depend on detailed patient assessment. Future directions for tablet use include increasing online access to the questionnaire, expanding use of the reports outside the clinic to other supportive services, and connecting the tablet to the electronic medical record. Adapting similar tools in the community would increase consistency and communication for patients and providers.

## Conclusions

In light of the increased incidence of cancer diagnoses and the growing population of cancer survivors, oncology practices must invest in tools that support patients, clinicians, and business operations. Incorporating the tablet into survivorship care is a successful way to meet many national standards surrounding cancer survivorship and quality care. Broadly speaking, this tool could help shape the future directions in cancer care, palliative care, and survivorship.
